# Lexibank, a public repository of standardized wordlists with computed phonological and lexical features

**DOI:** 10.1038/s41597-022-01432-0

**Published:** 2022-06-16

**Authors:** Johann-Mattis List, Robert Forkel, Simon J. Greenhill, Christoph Rzymski, Johannes Englisch, Russell D. Gray

**Affiliations:** 1 Department of Linguistic and Cultural Evolution, Max Planck Institute for Evolutionary Anthropology, Leipzig, Germany; 2 Institut für Orientalistik, Indogermanistik, Ur- und Frühgeschichtliche Archäologie, Friedrich-Schiller University, Jena, Germany; 3 ARC Centre of Excellence for the Dynamics of Language, Australia National University, Canberra, Australia; 4 School of Psychology, University of Auckland, Auckland, New Zealand

**Keywords:** Social anthropology, Interdisciplinary studies, Social anthropology

## Abstract

The past decades have seen substantial growth in digital data on the world’s languages. At the same time, the demand for cross-linguistic datasets has been increasing, as witnessed by numerous studies devoted to diverse questions on human prehistory, cultural evolution, and human cognition. Unfortunately, most published datasets lack standardization which makes their comparison difficult. Here, we present a new approach to increase the comparability of cross-linguistic lexical data. We have designed workflows for the computer-assisted lifting of datasets to Cross-Linguistic Data Formats, a collection of standards that make these datasets more Findable, Accessible, Interoperable, and Reusable (FAIR). We test the Lexibank workflow on 100 lexical datasets from which we derive an aggregated database of wordlists in unified phonetic transcriptions covering more than 2000 language varieties. We illustrate the benefits of our approach by showing how phonological and lexical features can be automatically inferred, complementing and expanding existing cross-linguistic datasets.

## Abstract (summary)

**Table:** 

Measurement(s)	expressions
Technology Type(s)	data aggregation
Factor Type(s)	none
Sample Characteristic - Organism	human language
Sample Characteristic - Location	global scale

## Background & Summary

Comparing the world’s languages opens new windows on human prehistory, culture, and cognition. By comparing languages historically, we can trace their evolution back in time and compare it with findings from archaeology and genetics^1,2^. By comparing languages typologically, we can learn about universal tendencies and cultural variation underlying the distribution of linguistic traits^3,4^ and investigate the degree to which linguistic trends are shaped by external factors^5,6^. By comparing linguistic findings across many languages with findings in cognitive science and psychology, we can foster a broader understanding of human cognition and behaviour^7–9^.

To compare the languages in the world, linguistic data must be assembled in a way that maximizes the comparability of individual data points across resources and language families. Although the amount of digitally available data for the world’s languages has been drastically increasing in the past decades^10^, the amount of comparable data is still relatively low. This problem is further heightened because more extensive collections of data compiled in the past have often not been archived for long-term durability. As a result, quite a few datasets have disappeared from the internet and are no longer available now^11,12^, although they played a substantial role in previous publications.

Inspired by the GenBank database^13^, where scholars can deposit nucleotide sequences publicly, we have created Lexibank, a collection of cross-linguistic datasets in standardized formats^14^, which offers access to word forms, sound inventories, and lexical features for more than 2000 language varieties derived from 100 individual high-quality datasets^15^.

The Lexibank wordlist collection is a first attempt to integrate the wealth of language data assembled during the past centuries. Although far away from being complete, we are convinced that the collection will provide a rich source for future investigations into the history, the diversity, and the psychology of the world’s languages.

There are numerous ways in which the Lexibank data can be analyzed and used. Assembling lexical data for a large number of languages, Lexibank offers multiple possibilities for researchers investigating cross-linguistic aspects of the lexicon of human languages. Thus, with respect to specific semantic domains, Lexibank allows scholars to expand previous studies on *color term evolution*^16^, *body part terminology*^17^, or *emotion semantics*^4^. With respect to the relation between lexical form and meaning, Lexibank offers the largest collection of lexical data with standardized transcriptions and semantic glosses, allowing scholars to test individual hypotheses on sound symbolism in the worlds’s languages^18^. With respect to the investigation of general aspects of lexical organization, Lexibank offers one of the largest cross-linguistic collections of form-meaning pairs, allowing scholars to search for various factors that shape the lexicon of the world’s languages^8^. For the purposes of historical language comparison, the Lexibank wordlist collection offers the largest assembly of expert judgements on historically related (cognate) words available to date. Given that computational methods for the detection of cognates are still not able to compete with experts^19^, our collection thus offers rich material to test and train new methods in the future. Similarly – given that the Lexibank collection unifies data on a global basis – scholars can use the data collection to test new methods for the automated identification of borrowings^20,21^, or to expand upon previous approaches to the automated detection of contact areas^22–24^. In addition, we illustrate how the data can be used to automatically extract various phonological and lexical features for individual language varieties.

By providing a detailed, replicable workflow through which lexical datasets in various formats can be unified and lifted to common standards, the Lexibank collection also contributes to increasing the ‘FAIRness’ of cross-linguistic datasets, by making data Findable, Accessible, Interoperable, and Reusable^25^, fulfilling the initial goal of the Cross-Linguistic Data Formats initiative^14^ and contributing to reproducible research in linguistics^26^.

Given the success of open, standardized data in evolutionary biology and genetics^27^, there is hope that increased future collaborative efforts in data standardization and curation could instigate a similar boom of new methods and insights in the language sciences. Our plan for the future is not only to expand this data collection further by contributing new datasets ourselves but also to encourage colleagues all over the world who collect cross-linguistic data to contribute to this ongoing endeavor and to share their data in an open, standardized form.

## Methods

### Background on cross-linguistic lexical datasets

*Structural datasets*, such as the World Atlas of Language Structures, are one key type of data used in cross-linguistic studies. (https://wals.info)^28^. Structural datasets assemble linguistic data points in the form of features that answer concrete questions on specific characteristics of a language. The questions can be directed to various linguistic domains, ranging from phonology (e.g. *Does the language have labiodental sounds?*)^6^, via syntax (e.g. *What is the language’s basic word order?*)^29,30^, and the lexicon (e.g. *Does the language use the same word to express ‘fear’ and ‘surprise’?*)^4^. The advantage of structural datasets is that individual features can be compared directly across languages and that the answers – which tend to be in numerical or categorical form – are usually straightforward to interpret. However, the disadvantage of structural datasets is that they are difficult to assemble – since linguists typically have to create them from dictionaries and reference grammars – and that their extraction is error-prone since it depends directly on human interpretation and analysis^31^.

Alternative forms of data applicable for cross-linguistic studies are *multilingual wordlists* and *parallel texts*. Wordlists offer translations for collections of concepts (typically reflecting vocabulary of everyday use) into various target languages. Parallel text collections provide translations of the same base texts into several languages. Both parallel texts and wordlists have been collected for a long time, since at least the late 18th century^32,33^. However, as automated text and sequence comparison methods require digital data, it has not been until recently that scholars started to employ them for large-scale cross-linguistic studies^34–36^.

Different attempts to assemble cross-linguistic wordlists have been made in the past. The Comparative Bantu OnLine Dictionary project (CBOLD, http://www.cbold.ish-lyon.cnrs.fr/), which started in 1994, represents one of the earlier born-digital efforts to present lexical data but has not been updated since 2000^37^. The PanLex project (https://www.panlex.org/) provides an extensive collection of Swadesh lists – wordlists that use concept lists originally compiled by Morris Swadesh as a questionnaire^38,39^ – for almost 2000 language varieties^40^.

The drawback of the collection is that its sources are not well documented, and forms are not provided in standardized phonetic transcriptions. The ASJP database (https://asjp.clld.org) is the most extensive wordlist collection in terms of cross-linguistic coverage, offering wordlists of about 40 items for more than 5000 language varieties in a unified phonetic transcription system^41^. The drawback of the ASJP database, however, is that the coverage in terms of concepts is very low, and even the goal of providing translations for a small list of 40 concepts is only met for about 86% of the varieties in the current version. Additionally, the transcription system merges many distinctions provided in the traditional International Phonetic Alphabet and therefore only offers limited possibilities for cross-linguistic studies on phonological variation.

In contrast, The Intercontinental Dictionary Series (IDS, https://ids.clld.org) has far fewer languages but a far larger concept list with translations of more than 1400 concepts into more than 300 language varieties^42^. Unfortunately, a major problem of the IDS is not its lack of cross-linguistic coverage but the fact that linguistic forms are not provided in unified phonetic transcriptions. As a result, the data can only be used for language-internal comparison such as the cross-linguistic investigation of colexification patterns^43^ where the same word form expresses multiple concepts in the same language^44^. Russian russianрука (*ruka*) typically refers to both ‘hand’ and ‘arm,’ reflecting a pattern that can be found in many of the world’s languages.

In addition to global wordlist collections, there are also extensive wordlist collections targeting specific linguistic “macro areas”, such as, for example, the NorthEuralex database (http://northeuralex.org), which offers standardized wordlists for more than 1000 concepts translated into more than 100 Eurasian languages^45^, or the Hunter-Gatherer database (https://huntergatherer.la.utexas.edu/), which assembles wordlists of varying size and structural features for more than 400 language varieties^46^. Table 1 provides an overview of major lexical databases that have been published in the past.

**Table 1:** Comparing lexical wordlist collections which have been published in the past decades.

Dataset	Source	Target Area	Concepts	Languages	Transcriptions
ABVD	Greenhill *et al*.^104^	Austronesian languages	210	>1000	—
ASJP	Wichmann *et al*.^41^	Global	40	>5000	custom
Chirila	Bowern 2016^105^	Australia	~300	>200	—
DIACL	Carling *et al*.^77^	Global	>400	>300	—
GLD	Starostin and Krylof 2011^106^	Global	110	>300	custom
HunterGatherer	Bowern *et al*.^46^	Australia and South America	>700	>400	—
IDS	Key and Comrie^42^	Global	1310	>300	—
NorthEuralex	Dellert *et al*.^45^	North Eurasia	1005	>100	IPA
Reflex	Ségerer and Flavier 2015^107^	African languages	from <100 to >1000	>300 (?)	—
STEDT	Matisoff 2015^108^	Sino-Tibetan languages	from <100 to >1000	>400 (?)	—
TransNewGuinea.org	Greenhill 2015^109^	New Guinea languages	from 40 to >700	>1000	—

Question marks in brackets after the record indicate that the total number of languages is not officially documented and therefore uncertain.

So far, the basic strategy of large-scale wordlist collections has been to assemble data language by language. Following language or area-specific documentation standards on concepts and orthographies, scholars seek to assemble as many wordlists for as many languages as possible, eventually reaching a point where it becomes more and more challenging to add more data or where a region has been sufficiently covered. Since collections will inevitably exploit existing datasets, the process of data collection involves a considerable amount of reformatting, adjusting, and modifying independently published datasets. This process bears the danger of introducing errors into the derived data, especially when a source is interpreted and converted to adjust it to the new resources. Another problem arises from the lack of flexibility in closed data collections with a fixed number of concepts and a fixed phonetic transcription system. Since decisions to ignore or recode parts of the original data during data collection cannot be easily reverted, data collections often omit more significant pieces of the original information from which they are drawn.

An alternative to assembling data language by language consists of *lifting* individual datasets to common standards from which custom data collections can be later aggregated. For this strategy, the availability of *reference catalogues* (which describe basic linguistic constructs, such as language varieties, concepts, and speech sounds) and standard formats for data exchange (table structures, metadata) is crucial. Initial ideas to address the problem resulting from the lack of standards and exchange formats for cross-linguistic data were presented as part of the Cross-Linguistic Data Formats (CLDF) initiative (https://cldf.clld.org^14^). CLDF offered first specifications for wordlists and structural datasets and outlined how cross-linguistic lexical and structural data can standardized and how software packages can help to validate if data conforms to the newly proposed standards. Building on CLDF, we have developed improved ways to convert cross-linguistic lexical data into the new standards. We have tested these workflows by lifting various datasets published during the past decades and by entertaining collaborations with active data collectors. In sum, this collection, which we call Lexibank, consists of 100 individual CLDF datasets covering more than 4000 wordlists from more than 2400 language varieties. To illustrate the interoperation and reuse potential of this data collection, we develop a new suite of software tools that allow us to extract various phonological and lexical features from the data automatically.

### Cross-linguistic data formats

The CLDF initiative was initially launched in 2014 by researchers from different institutions to address common reuse and portability problems of digital cross-linguistic data^47^. The solution proposed by the CLDF initiative was to unify cross-linguistic datasets by proposing relatively straightforward tabular formats for the representation of lexical, structural, and parallel text data^14^. While earlier standardization efforts often strived for completeness (in the sense of *expressive adequacy*^48^), CLDF chose computational reusability as the primary design goal. Thus, the CLDF specification is comparatively small but comes – by design – with clear examples showing how the data could be analyzed computationally^49^. From 2018 on, we further refined the original specifications by expanding the specification to account more properly for phonetic transcriptions. An important step was the integration of the extended standards for phonetic transcriptions provided by Cross-Linguistic Transcription Systems (CLTS, https://clts.clld.org), a reference catalog that maps phonetic transcriptions to speech sounds^50,51^. In the last two years, all three major reference catalogs referenced by CLDF – Glottolog for languages^52^, Concepticon for concepts^53^, and CLTS for speech sounds – were drastically refined in order to allow for a more detailed integration of cross-linguistic data. First attempts were carried out to model additional cross-linguistic data types, such as interlinear-glossed text^54^. Details of this process can be found on the project website of the CLDF initiative (https://cldf.clld.org). For future refinements of CLDF, we have adopted the practice to present them first in dedicated studies along with examples and then discuss whether to integrate them in subsequent new releases of the CLDF specification^55^.

### (Retro-)Standardization of lexical datasets

The standardization of lexical datasets with the help of CLDF comes in two forms. First, CLDF can be used to increase the comparability of existing datasets in the form of retro-standardization. Second, CLDF can be used during the process of data collection and curation to provide consistency checks of the raw linguistic data. In order to enhance both forms of standardization, we created the PyLexibank Python package^56^ on top of the generic CLDFBench package^57^. CLDFBench allows users to convert their data with a few lines of code to CLDF formats, but lacks specific solutions that are important for the creation of lexical data. PyLexibank builds on CLDFBench to allow for a facilitated and more targeted curation of lexical data by providing integrated support of the Concepticon^53^ and the CLTS reference catalogs^58^. The primary service offered by the PyLexibank package is an explicit integration of the reference catalogs, which are important to make lexical data comparable, namely Concepticon, for the standardization of concept identifiers, derived from elicitation glosses in lexical wordlists^59^, and CLTS for the standardization of phonetic transcriptions^50^.

The linking of lexical data to the Concepticon project is organized in a dedicated workflow maintained by the editorial team of the Concepticon project. The workflow has been described in detail in previous studies^60–62^. The conversion of phonetic transcriptions to the standards provided by the CLTS project are organized with the help of orthography profiles^63^. Orthography profiles are straightforward lookup-tables which define individual graphemes in a given orthography (a grapheme being a unit consisting of one or more characters) along with their target value in the standardized transcription system. PyLexibank facilitates the creation and curation of orthography profiles by allowing users to create a draft profile from their raw data. It uses a method for the automatic segmentation of phonetic transcriptions originally designed for the LingPy software package^64^. In this way, a first ‘draft profile’ can be created, which users can then refine systematically. The PyLexibank package offers additional routines to pre-process lexical forms with general cleaning routines (stripping off brackets, splitting entries, etc.). Having refined the profile, the data can be segmented with the Segments package^65^ and verified with the PyCLTS package^66^. Details of the process of orthography profile creation have been discussed in previous studies^67,68^. Table 2 summarizes the basic operations. How the software packages upon which the Lexibank repository builds are integrated and applied in practice has been documented in several hands-on tutorials by team members and early adopters who illustrate how datasets can be lifted to CLDF and added to the Lexibank repository^69,70^.

**Table 2:** Basic operations involving the lifting of data to the CLDF standards with the help of the PyLexibank package.

Procedure	Reference Catalog	Software	Description
link languages	Glottolog	PyGlottolog	Link the language names to the identifiers provided by the Glottolog reference catalog. Currently, this is done manually in most parts.
map concepts	Concepticon	PyConcepticon	Map elicitation glosses in the original wordlist data to the concept identifiers provided by the Concepticon reference catalog. Software for semi-automated concept mapping is used for this task and then manually refined.
unify transcriptions	CLTS	PyLexibank LingPy Segments PyCLTS	Unify transcription systems by converting the transcriptions to the standards provided by the CLTS reference catalog. This procedure is by far the most complex one, which involves the cleaning of lexical forms, using dedicated routines in the PyLexibank package, the creation of a draft profile with the help of the LingPy package, the manual refinement of the profile and its application with the help of the Segments package, and finally its verification with the help of the PyCLTS package.

### Automatic feature extraction

Although language features are often defined differently, basic feature types can easily be identified and often even computed in a common fashion. Similar to the process of *feature aggregation* underlying the AUTOTYP database for structural features^71,72^, we offer computational methods to extract phonological and lexical features from the Lexibank wordlist collection. For example, consider the feature ‘Consonant Size’, which comprises the number of consonants in a given language. Once data are provided in a wordlist in phonetic transcription and segmented in such a way that unique sounds can be identified, a lower bound for the number of consonants in a given language can be approximated by counting the distinct sounds in the wordlist sample. Although this approach may fail to elicit all consonants since there is no guarantee that a smaller collection of words will contain all sounds in a language^73^, it approximates the real number of sounds reasonably well. Since all data in the LexiCore subset of our Lexibank collection are linked to the sound identifiers provided by the CLTS project^58^, which in turn each define a sound by a bundle of distinctive features, we can easily extract additional subsets of sounds depending on their distinctive features. In this way, our code for feature extraction, which is implemented as part of a dedicated software package (CL Toolkit, https://pypi.org/project/cltoolkit^74^), defines various features by means of straightforward software operations. These operations check if subsets of sounds in the sound inventory of a given language have a certain feature or a certain combination of features (see Section *Technical Validation*).

Some phonological features, like the features on prosody or sound symbolism, require additional data or functions. Prosodic features computed by CL Toolkit, for example, make use of an automatic syllabification procedure based on the sonority of individual sounds^75^ implemented by the LingPy software package^64^. Features on sound symbolism, which are determined by checking if a word expressing a certain concept has certain phonetic properties, additionally need to take information from the Concepticon reference catalog into account^53^, which standardizes concepts in the Lexibank collection.

The extraction of lexical features checks for the full or partial identity of the word forms expressing dedicated concepts. Thus, in order to check whether ‘arm’ and ‘hand’ are colexified in a given language, the method first looks up the Concepticon Concept Sets ‘ARM’ 1637, ‘HAND’ 1277, and ‘ARM OR HAND’ 2121 and then checks whether word forms for ‘ARM’ and ‘HAND’ are present and if so, if they are identical. If they are identical, it identifies a colexification, if not, it checks if a word form for ‘ARM OR HAND’ is present, which would entail the colexification, identifying a colexification if this is the case or otherwise yielding a negative result. In a similar way, the method checks for the existence of common substrings or affix colexifications.

The code for the automatic extraction of phonological and lexical features is written in such a way that users can expand it easily in the future. Since the entities from which the features are extracted are standardized descriptors for sounds or concepts, extensions of our current code base can be easily written and integrated or applied by creating light-weight plugins to our current solutions provided in the CL Toolkit package.

## Data Records

### Lexibank wordlist collection

Lexibank^15^ is a meta-collection of standardized wordlists compiled from various individual datasets. The standardized wordlists themselves are independently curated. Their curation follows the data curation workflow of the Lexibank project, which uses the PyLexibank Python library^56^ to convert lexical data in custom formats into CLDF wordlists. The editorial board of the Lexibank project decides about the inclusion of individual datasets into the Lexibank wordlist collection. Datasets which are included in this collection need to be archived with Zenodo (https://zenodo.org/) and curated in a GIT repository (https://git-scm.com/). Datasets included into the Lexibank wordlist collection are referenced with their Zenodo DOI and the URL of their GIT repository and classified for their level of standardization (file etc/lexibank.csv in the Lexibank repository.

The Lexibank wordlist collection is provided in the form a CLDF dataset itself. The dataset is augmented by Python code which can be called from the commandline and allows users to download all individual datasets from their archives (Zenodo and GitHub). In addition, the code allows to compute phonological and lexical features from the data and store them in CLDF formats. All individual wordlists referenced in the Lexibank repository as well as the Lexibank repository itself are licensed under a Creative Commons 4.0 License. The Lexibank repository is curated on GitHub (https://github.com/lexibank/lexibank-analysed) and archived with Zenodo (10.5281/zenodo.5227817)^15^. The current release of the repository is Version 0.2.

Lexibank (version 0.2) currently assembles lexical data from 100 different datasets which together offer wordlists for 4069 language varieties, corresponding to 2456 distinct languages and dialects (as identified by Glottolog^52^), and providing information for a total of 3110 lexical concepts, with a total of 1,912,952 words. Wordlists in the Lexibank collection show different degrees of standardization representing the level to which they can be lifted. For 3320 wordlists taken from 94 datasets, fully standardized phonetic transcriptions can be provided for at least 80 word forms. We call this dataset the LexiCore subset of Lexibank (see dataset chingelong^76^ for an example). For 1806 wordlists from 52 datasets, large wordlists of at least 250 standardized concepts can be provided, but individual wordlists do not necessarily all offer fully standardized phonetic transcriptions. We call this dataset the ClicsCore subset of Lexibank (see dataset diacl^77^ for an example). 1441 wordlists from 49 datasets are not only available in standardized phonetic transcriptions but also offer information on etymologically related words (cognate sets) provided by experts. We call this dataset the CogCore subset of Lexibank (see dataset liusinitic^78^ for an example). A small subset of 18 wordlists from 4 datasets even offers proto-forms – forms inferred for unattested ancestral languages, using the traditional techiques of the comparative method^79^ – in standardized phonetic transcriptions. This dataset is called the ProtoCore subset of Lexibank (see dataset davletshinaztecan^80^ for an example).

**Fig. 1:**
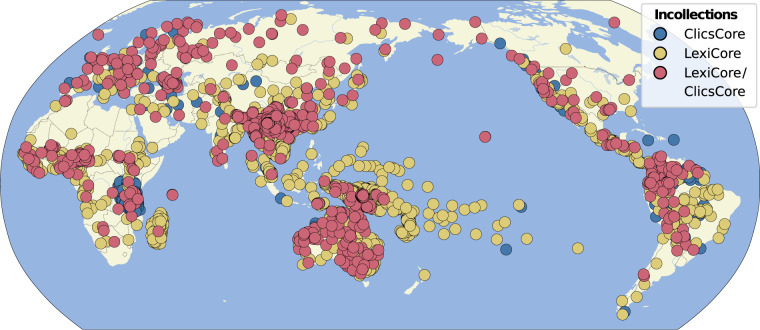
Distribution of lexical resources with phonetic transcriptions (LexiCore) and lexical resources with a larger number of lexical forms (ClicsCore) in the Lexibank wordlist collection.

Figure 1 shows the distribution of the data for the LexiCore (wordlists with standardized transcriptions) and the ClicsCore (wordlists with large coverage in terms of concepts) wordlists in our collection. While we can see that some regions of the world are less well covered than others, we can also see that the current collection has already reached a considerable worldwide coverage. Table 3 provides general statistics on the datasets assembled as part of the Lexibank collection.

**Table 3:** Comparing lexical wordlist collections which have been published in the past decades.

ID	Name	Description	Datasets	Varieties	Glottocodes	Concepts	Forms
Lexibank	all wordlists in the Lexibank collection	Metacollection of wordlists belonging to either of the datasets.	100	4069	2456	3110	1,912,952
LexiCore	wordlists with phonetic transcriptions	Wordlists with phonetic transcriptions in which sound segments can be readily described by the CLTS system.	94	3320	2208	3050	1,041,766
ClicsCore	large wordlists with at least 250 concepts	Wordlists with large form inventories in which at least 250 concepts can be linked to the Concepticon.	52	1806	1098	3043	1,496,855
CogCore	wordlists with phonetic transcriptions and cognate sets	Wordlists with phonetic transcriptions in which cognate sets have been annotated (a subset of LexiCore).	49	1441	1114	1670	275,249
ProtoCore	wordlists with phonetic transcriptions, cognate sets, and proto-languages	Wordlists with phonetic transcriptions in which cognate sets have been annotated and which contain one or more ancestral languages whose forms are proto-forms from which forms in the descendant languages can be derived (a subset of CogCore).	4	18	18	951	8,750

### Collection of phonological and lexical features

**Fig. 2:**
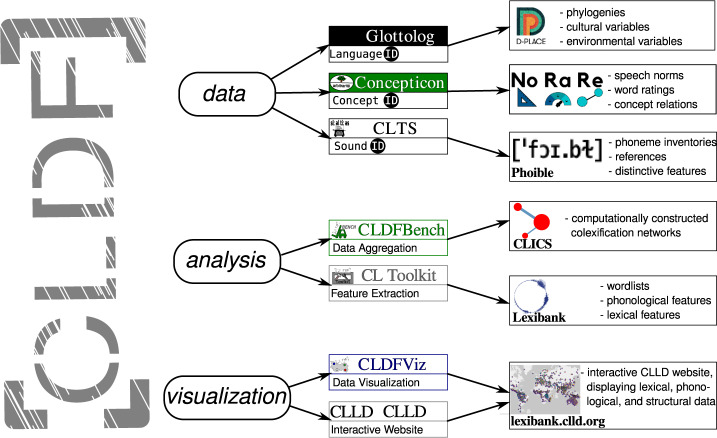
Reference catalogs, tools for analysis, and tools for visualization, integrated by CLDF datasets. By providing active links to the identifiers of Glottolog, Concepticon, and by converting phonetic transcriptions to the standard transcriptions provided by the CLTS catalog, CLDF datasets can be integrated with other existing datasets, such as D-PLACE^81^, NoRaRE^62^, and PHOIBLE^89^. With the help of dedicated packages for the analysis of CLDF datasets, data can be easily aggregated with CLDFBench^57^, and features can be automatically extracted with the help of CL Toolkit^74^. For the visualization of CLDF datasets, data can be plotted on geographic maps with the help of CLDFViz^91^ and shared on interactive websites with the help of CLLD^110^.

The Lexibank data collection provides data in formats that facilitate both the *aggregation* of lexical data from different sources and the *integration* of aggregated data with other kinds of linguistic and non-linguistic information. Integration is guaranteed via the standards enforced by the CLDF specification and by reference catalogs, which provide large collections of metadata for standard constructs in linguistic research, such as languages (Glottolog^52^, https://glottolog.org), concepts (Concepticon^53^, https://concepticon.clld.org), and speech sounds (Cross-Linguistic Transcription Systems, CLTS^58^, https://clts.clld.org). Since all reference catalogs provide additional rich information on the linguistic constructs they define, linking data to reference catalogs allows to enrich existing datasets drastically. Furthermore, since the object identifiers (for languages, concepts, speech sounds) provided by the reference catalogs can be integrated in any additional resource, there are numerous ways to integrate the data further. Via Glottolog’s language identifiers, for example, cultural data from the D-PLACE^81^ database can be compared with lexical data in our Lexibank collection. Via the Concepticon’s concept identifiers, various kinds of speech norms, ratings, and conceptual relations can be retrieved via the cross-linguistic database of Norms, Ratings, and Relations (NoRaRe^62^, https://digling.org/norare/) database. Via the sound identifiers of the CLTS catalog, information on sound inventories from numerous sound inventory databases can be retrieved and compared^82^. Figure 2 illustrates how data provided in CLDF formats can be integrated by expanding the basic data with the help of reference catalogs, and by analyzing and visualizing the data with the help of dedicated software tools.

In addition to referencing datasets which provide wordlists in standards conforming to the Lexibank standards of data curation and data integration, the Lexibank reference catalog provides a collection of phonological and lexical features which were automatically extracted from the wordlist data. The computation makes use of the CL Toolkit Python package^74^ and can be invoked via the commandline as part of Lexibank’s workflow for data aggregation and data curation. The resulting feature collections provide automatically extracted phoneme inventories and phonological features for all language varieties in the LexiCore subset of Lexibank as well as automatically extracted lexical features for all language varieties in the ClicsCore subset. The feature collections are themselves stored in CLDF format and shared and archived with each release of the Lexibank repository.

## Technical Validation

Due to the high level of integration and standardization of wordlists, the Lexibank collection has a high potential for reuse. The data can be used as the starting point for various phylogenetic studies of individual language families. Given the large number of datasets in which etymological word relations across languages have been annotated by experts, the data can also serve as a benchmark to advance the development of new methods for automatic word comparison^19^ and automatic cognate word prediction^83,84^, which drastically exceeds the size of previously published benchmark datasets^85^. In addition, the data can be used to compute various kinds of phonological and lexical features for individual language varieties and thus actively contribute to future studies on linguistic diversity, human prehistory, and human cognition. In the following, we will concentrate on this last aspect and show how phonological and lexical features can be automatically computed from the Lexibank collection. In this way, we contribute to recent attempts to increase the transparency of cross-linguistic collections of structural data. We expect that the role which the formal extraction of discrete and continuous features from language data plays at the moment will gain much more importance in the future.

### Inference of phonological features

In comparative linguistics, various kinds of phonological features have been used in the past in order to compare languages. Phonological features comprise various characteristics related to the sounds of spoken languages or their combination, ranging from discrete features such as the phoneme size, reflecting the number of distinct sounds in a given language^86,87^, via continuous features, such as the ratio of consonant and vowel size^82^, and categorical features, such as the presence and type of lexical tone in a language^5^, up to binary features, such as the presence of labiodental sounds^6,88^. They are typically collected by extracting the relevant information directly from the linguistic literature (reference grammars, phonological descriptions, grammar sketches).

Since the LexiCore collection of the Lexibank wordlist collection contains word forms in standardized phonetic transcriptions, a great deal of phonological features can be automatically computed from the data. This has three major advantages. First, it saves a lot of time and labor because the feature extraction can be done automatically. Second, it increases the flexibility of feature annotation, since we are not bound to decide on one representation (categorical, continuous, etc.) of feature values before starting to collect the data but can experiment with different representations when designing methods for feature inference. Third, it is much more transparent as inferred features can be directly validated by referring back to the original data.

Our workflow for the extraction of phonological features from the wordlist in our LexiCore collection of Lexibank currently allows us to compute 30 distinct phonological features. Some of the features are also offered by large structural datasets^28^ and can be directly compared with them, while other features have not been assembled in publicly available datasets so far and may therefore offer interesting insights to language typologists.

Table 4 shows the 30 phonological features which we automatically extracted from the data. As can be seen from the table, the features can be classified into four distinct groups. There are discrete features on sound inventory sizes (1–7, number of vowels, consonants, etc.), there are various features on special sound types or individual specific sounds (8–19), there are three prosodic features (20–22), and eight features pertaining to specific sound-meaning relations (also termed “sound symbolism”, 23–30).

**Table 4:** Phonological features automatically extracted from the LexiCore data in Lexibank.

No.	Identifier	Name	Type
1	ConsonantQualitySize	consonant quality size	inventory size
2	VowelQualitySize	vowel quality size	
3	VowelSize	vowel size	
4	ConsonantSize	consonant size	
5	CVRatio	consonant and vowel ratio	
6	CVQualityRatio	consonant and vowel ratio (by quality)	
7	CVSoundRatio	consonant and vowel ratio (including diphthongs and clusters)	
8	HasNasalVowels	has nasal vowels or not	special vowels
9	HasRoundedVowels	has rounded vowels or not	
10	VelarNasal	has the velar nasal (engma)	
11	PlosiveVoicingGaps	voicing and gaps in plosives	
12	LacksCommonConsonants	gaps in plosives	
13	HasUncommonConsonants	has uncommon consonants	
14	PlosiveFricativeVoicing	voicing in plosives and fricatives	
15	UvularConsonants	presence of uvular consonants	
16	GlottalizedConsonants	presence of glottalized consonants	
17	HasLaterals	presence of lateral consonants	
18	HasLabiodentalFricatives	inventory has labio-dental fricatives or affricates	
19	HasPrenasalizedConsonants	inventory has pre-nasalized consonants	
20	SyllableStructure	complexity of the syllable structure	prosody
21	SyllableOnset	complexity of the syllable onset	
22	SyllableOffset	complexity of the syllable offset	
23	FirstPersonWithM	fist person starts with an m-sound	sound symbolism
24	FirstPersonWithN	fist person starts with an n-sound	
25	SecondPersonWithT	second person starts with a t-sound	
26	SecondPersonWithM	second person starts with an m-sound	
27	SecondPersonWithN	second person starts with an n-sound	
28	MotherWithM	mother starts with m-sound	
29	FatherWithP	father starts with p-sound	
30	WindWithF	wind starts with f-sound	

The detailed values which the features can take, are provided in the online documentation of the CL Toolkit package (https://cltoolkit.readthedocs.io/).

In order to evaluate the usefulness of our approach for automatic feature extraction from lexical datasets, we compare how well the inferred values for five selected features in LexiCore correlate with the features provided in the WALS database^28^ and the features inferred from PHOIBLE^89^. As can be seen from the results of this comparison in Table 5, our approach receives reasonably high correlations with both the features in WALS and those extracted from PHOIBLE, although PHOIBLE and WALS generally show a higher correlation with each other. This is, however, not surprising, given that both datasets are based on very similar sources by the same contributor (a larger part of PHOIBLE was taken from the UCLA Phonological Segment Inventory Database^90^, whose author Ian Maddieson also contributed the chapter on phonology in WALS, see the detailed study by Anderson *et al*.^51^ for a detailed discussion of the comparison of phoneme inventory database).

**Table 5:** Spearman rank correlation (*ρ*) coefficients of feature values in WALS, Phoible and LexiCore, for five selected features, calculated for those parts of the data where information in all three dataset could be obtained, matching languages by their common Glottocodes.

Feature	WALS/LexiCore	WALS/Phoible	LexiCore/Phoible	Sample
ConsonantSize	0.66/*p* < 0.01	0.92/*p* < 0.01	0.70/*p* < 0.01	233
VowelQualitySize	0.51/*p* < 0.01	0.66/*p* < 0.01	0.68/*p* < 0.01	235
CVRatio	0.55/*p* < 0.01	0.76/*p* < 0.01	0.68/*p* < 0.01	235
PlosiveFricativeVoicing	0.54/*p* < 0.01	0.69/*p* < 0.01	0.59/*p* < 0.01	235
PlosiveVoicingGaps	0.40/*p* < 0.01	0.60/*p* < 0.01	0.56/*p* < 0.01	235

When more than one language was available for the same Glottocode, the median value was taken.

**Fig. 3:**
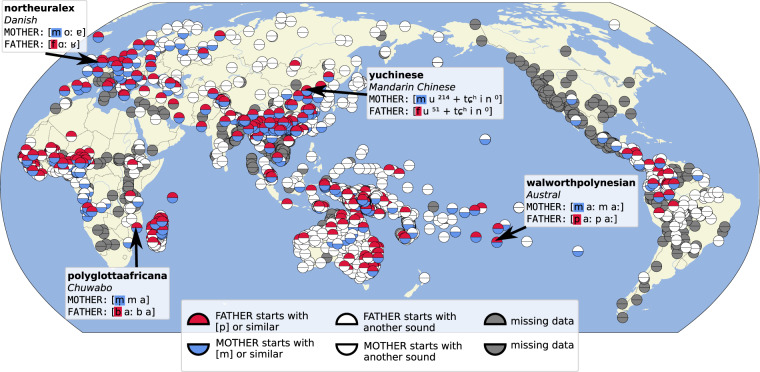
Comparing cross-linguistic patterns of sound symbolism involving words for ‘mother’ and ‘father’ in the world’s languages. The four datasets from which the four examples showing actual forms for individual language varieties are taken are indicated in the figure.

Investigating the features inferred with our workflows requires tools for exploratory data analysis. One way to explore large feature collections for cross-linguistic data is to plot them on a geographic map in order to see whether specific areal patterns emerge. CLDF comes with a dedicated suite of software tools for data visualization which greatly facilitate this part (CLDFViz^91^), allowing users to create high-quality static and interactive maps in which features can be combined ad libitum. An example for such a map is shown in Fig. 3, where we have plotted the features 28 and 29 in our collection, which ask whether words for ‘mother’ and ‘father’ start with [m] and [p] respectively, reflecting a well-known trend that can be observed in the world’s languages and is usually attributed to the sounds children learn during first-language acquisition^92^. As can be seen from the map, our data confirms the global trend. Many unrelated languages spoken in different geographic areas have words for ‘mother’ which start with [m] and words for ‘father’ which start with [p] or similar sounds (including labiodental fricatives like [f]). More detailed investigations would require in-depth analyses by language typologists, for which our dataset provides a useful starting point.

### Inference of lexical features

Languages differ in the way in which their lexicons are structured. One of the most prominent aspects in which languages differ is to which degree they use the same word forms to denote different concepts. Russian *ruka*, for example, can mean ‘arm’ and ‘hand,’ and German *Decke* can mean ‘ceiling’ and ‘blanket.’ This phenomenon, termed colexification in the recent linguistic literature (a cover term for polysemy on the one hand and homophony on the other hand^44^), has recently received broader attention among linguists^93^, psychologists^4^, and computer scientists^94^, and is most prominently represented in the Database of Cross-Linguistic Colexifications (CLICS, https://clics.clld.org^60,61,95^) which aggregates colexifications from CLDF datasets for more than 2000 language varieties. While the original CLICS database was built from 30 datasets, the ClicsCore collection in Lexibank expands this collection by 20 additional datasets. Retaining only those languages which provide at least 250 concepts which can be linked to the Concepticon reference catalog, ClicsCore contains 1806 different language varieties corresponding to 1114 different languages (as reflected by unique Glottocodes in the Glottolog reference catalog).

While the original CLICS data identifies only those cases as colexifications where an identical word form denotes two different senses, we expand the notion of colexification in our feature extraction procedure by adding two more types of colexification which have so far only been sporadically discussed in the literature. First, we add a method for the identification of partial colexifications, defined as those cases in which two word forms expressing two different concepts are not identical, but share a common substring, and affix colexifications, where one word appears as a prefix or a suffix of another word (see Table 6 for examples and full definitions). Searching systematically for these colexifications in our data allows us to identify commonalities in the languages of the world and to investigate whether they are due to areal proximity, common descent, or rather general cognitive principles.

**Table 6:** Colexification patterns that can be computed from the ClicsCore subset of the Lexibank wordlist collection.

Type	Description	Examples
full colexification	Two different senses are expressed by the same word form.	Russian *ruka* ‘hand’ vs. *ruka* ‘arm’.
		German *Decke* ‘blanket’ vs. *Decke* ‘ceiling’.
partial colexification	Two word forms expressing two different senses are expressed by word forms which share a common substring	German *be-****antwort****-en* ‘answer’ vs. *ver-****antwort****-en* ‘be responsible’.
affix colexification	Of two word forms expressing two different senses, one word form is identical with the beginning or the end of the other word form.	German *Fingernagel* ‘fingernail’ vs. *Nagel* ‘nail (tool)’.
		German *Ellenbogen* ‘elbow’ vs. *Bogen* ‘bow (arc)’.

The 30 features which we compute from the ClicsCore subset of our wordlist collection are given in Table 7. While we could easily expand this collection further, we have limited the features to those cases which have been previously discussed in the literature and collected manually in structural datasets.

**Table 7:** 30 lexical features which can be automatically extracted from the ClicsCore subset of Lexibank.

No.	Identifier	Name	Type
1	LegAndFoot	has the same word form for foot and leg	colexification
2	ArmAndHand	arm and hand distinguished or not	
3	BarkAndSkin	bark and skin distinguished or not	
4	FingerAndHand	finger and hand distinguished or not	
5	GreenAndBlue	green and blue colexified or not	
6	RedAndYellow	red and yellow colexified or not	
7	ToeAndFoot	toe and foot colexified or not	
8	SeeAndKnow	see and know colexified or not	
9	SeeAndUnderstand	see and understand colexified or not	
10	ElbowAndKnee	elbow and knee colexified or not	
11	FearAndSurprise	fear and surprise colexified or not	
12	CommonSubstringInElbowAndKnee	elbow and knee are partially colexified or not	partial colexification
13	CommonSubstringInManAndWoman	man and woman are partially colexified or not	
14	CommonSubstringInFearAndSurprise	fear and surprise are partially colexified or not	
15	CommonSubstringInBoyAndGirl	boy and girl are partially colexified or not	
16	EyeInTear	eye partially colexified in tear	affix colexification
17	BowInElbow	bow partially colexified in elbow	
18	CornerInElbow	corner partially colexified in elbow	
19	WaterInTear	water partially colexified in tear	
20	TreeInBark	tree partially colexified in bark	
21	SkinInBark	skin partially colexified in bark	
22	MouthInLip	mouth partially colexified in lip	
23	SkinInLip	skin partially colexified in lip	
24	HandInFinger	hand partially colexified in finger	
25	FootInToe	foot partially colexified in toe	
26	ThreeInEight	three partially colexified in eight	
27	ThreeInThirteen	three partially colexified in thirteen	
28	FingerAndToe	finger and toe colexified or not	
29	HairAndFeather	hair and feather colexified or not	
30	HearAndSmell	hear and smell colexified or not	

Features can be divided into three major classes, depending on the type of colexification they reflect: (A) colexifications, referring to cases of polysemy in which one word form expresses two distinct senses, (B) partial colexification, referring to cases in which two word forms expressing distinct senses share a common substring, and (C) affix colexification, referring to cases in which one word form starts or ends with another word form.

**Fig. 4:**
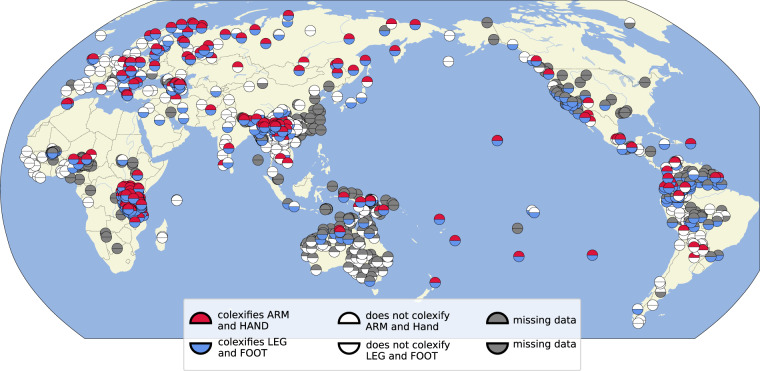
Global distribution of languages in the ClicsCore subset of Lexibank which colexify ‘arm’ and ‘hand’ and ‘leg’ and ‘foot’ respectively.

As a first example for the potential of large aggregated datasets, Fig. 4 shows which languages in our collection colexify ‘arm’ with ‘hand’ and ‘leg’ with ‘foot,’ respectively. Previous studies have almost exclusively concentrated on the global distribution of languages colexifying ‘arm’ and ‘hand,’ assuming that there is a geographic tendency to colexify the terms more frequently, the closer one comes to the equator^96^. Contrasting the colexification pattern with its logical counterpart yields interesting patterns, in so far, as our analysis suggests a rather strong systemic tendency across languages from different language families and areas to either express both ‘arm/hand’ and ‘foot/leg’ by the one word each, or to distinguish them both. More research on this topic is needed. The data we have assembled here are a helpful starting point.

**Fig. 5:**
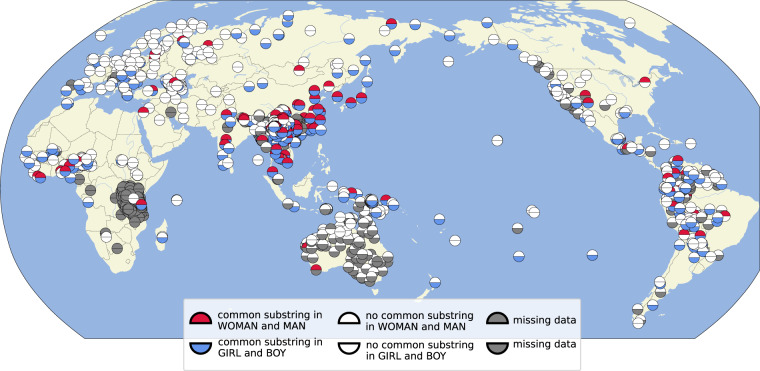
Partial colexifications between ‘woman’ and ‘man’ and between ‘daughter’ and ‘son’.

Figure 5 provides another example on features which partially occur in correlated form. This time, we compare whether languages denote ‘woman’ and ‘man’ by means of a partial colexification (compare 女人 *nǚ-rén* ‘female person → woman’ vs. 男人nan-ren ‘male person → man’ in Mandarin Chinese) on the one hand, and ‘daughter’ and ‘son’ (compare 女兒 *nǚ-*ě*r* ‘female offspring → daughter’ vs. 兒子 ě*rzĭ* ‘offspring-son → son’) on the other hand. The analysis suggests a large areal cluster in South-East Asia, where the tendency of languages to use compound words in a rather analytical manner is well known, as well as some languages in the North of South America, but the pattern shows a less global distribution than the one for ‘arm’ vs.‘leg’ shown in Fig. 4.

**Fig. 6:**
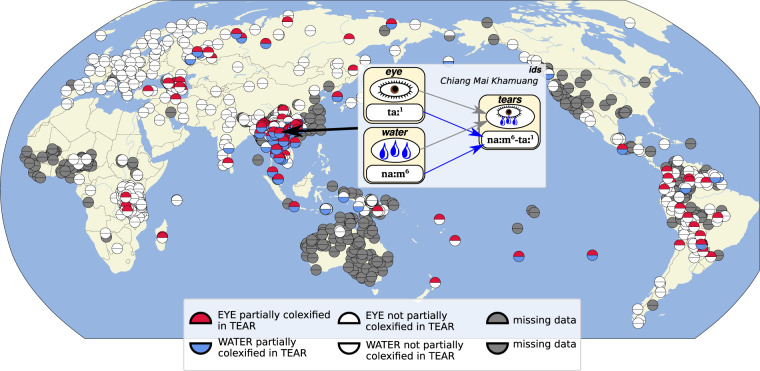
Comparing which languages express ‘tear’ as ‘eye-water’ in the ClicsCore sample of Lexibank.

As a final example, Fig. 6 compares affix colexifications in which words recur in the beginning of another word, indicating strong semantic relations. In the concrete example, we check to which degree the word for ‘tear’ in the languages in our sample is composed of the word for ‘eye’ and the word for ‘water’ respectively. That ‘tears’ are denoted as ‘eye-water’ is a common pattern that can be found in quite a few South-East Asian languages (compare Younuo [ki^55^ mo^32^-ʔŋ^44^] ‘eye-water’^68,97^), but also in a few languages in South America (compare Guaraní *esa-ɨ* ‘eye-water’)^42^. As can be seen from the Figure, we find that South-East Asian languages indeed overwhelmingly express ‘tears’ as ‘eye-water,’ in so far as they show an affix colexification of ‘eye’ and of ‘water’ with ‘tear,’ but apart from this, the feature only occurs sporadically.

## Usage Notes

### Distribution of lexibank datasets

For the distribution of CLDF datasets in general and Lexibank datasets in specific, we use existing long-term archiving solutions provided by Zenodo (https://zenodo.org). Once a Lexibank dataset has been created and the creators consider the data ready to be shared publicly, a new version of the data is created and archived with Zenodo, using the automated integration of Zenodo with GitHub. In addition, the new version is tagged as part of the Lexibank community on Zenodo (https://zenodo.org/communities/lexibank), which allows users to browse conveniently through the large collection of available datasets. Zenodo is a partner of OpenAIRE (https://www.openaire.eu/) and indexed by re3data (https://www.re3data.org) – and eventually by search engines like *Google Dataset Search*, thus addressing the *findability* problem of academic resources^98^.

### Promotion of lexibank

Lexibank and lexical data in CLDF formats have been promoted in several ways. First, we have conducted detailed studies in which CLDF formats are used along with CLDFBench and the PyLexibank software package, illustrating how data aggregation can be successfully carried out^60,61^, or showing how data can be supplemented in transparent CLDF formats^21,68^ Second, we have created certain flagship projects which showcase specific aspects of CLDF and the advantage of using integrated data^99,100^. Third, we have conducted projects with students and young scholars, who were trained to use our new resources and encouraged to share their knowledge in the form of small blog posts (published at https://calc.hypotheses.org) along with new datasets which bachelor, doctoral, and master students lifted themselves assisted by our team^70,101–103^.

Lexibank is an ongoing, collaborative effort and the participation of the wider community is very welcome. Our team of core contributors provides active support to those who want to learn how to prepare their data for inclusion in Lexibank. While proper inclusion of a dataset in a Lexibank release requires inclusion in the Lexibank community on Zenodo (https://zenodo.org/communities/lexibank), the free availability of the relevant software and the CLDF standard make it possible to combine external – or even private – data with Lexibank. Hopefully, this low bar for engaging with Lexibank as data consumer as well as data producer will foster a vibrant community.

## Data Availability

The main software package underlying Lexibank is curated on GitHub (https://github.com/lexibank/lexibank-analysed/tree/v0.2) and archived with Zenodo (10.5281/zenodo.5227817)^15^. Individual datasets belonging to the Lexibank wordlist collection are curated on individual repositories on GitHub (see our master list at https://github.com/lexibank/lexibank-analysed/blob/v0.2/etc/lexibank.csv) and are also all archived with Zenodo (see https://zenodo.org/communities/lexibank/).
